# Verminous Persons

**Published:** 1897-07-17

**Authors:** 


### The Hospital

A Journal of

The Medical Sciences and Hospital Administration.

Vol. XXII.—No. 564.] [July 17, 1897.

### Verminous Persons.

The House of Commons has lately been dealing somewhat playfully with a Bill for the relief of "Verminous Persons," by which it was proposed to give power to local authorities to take measures for the relief of those who were thus afflicted, but with the fatal defect that no fund was indicated from which the necessary expenditure might be defrayed. The conditions of the debate were governed by the circumstance that the measure next in order was the Female Suffrage Bill, which, by some inconceivable mismanagement, had previously been read a second time, and which it was thought expedient, by most of those who had voted for it, to keep at arm's length until the period at which it could be further considered had passed beyond recall. We do not wish to express any opinion, one way or the other, on the desirability of extending the suffrage to women; but it is at least certain that this will never be done except by a Bill brought forward or supported by the Government of the day, and exhaustively debated in all its stages. A few members of the House of Commons had been so left to themselves as to give hustings pledges of manifest insincerity in favour of the measure; and were therefore obliged to vote for it when, somewhat to their dismay, it was brought forward as an actual proposal for legislation. The only method of avoiding self-stultification was to take care that the Bill embodying it should not come before the House again, and the only method of effecting this object was to talk about the preceding Bill until the time at the disposal of the House was exhausted. This preceding Bill chanced to be that to which we have referred, and hence it befell that the smaller wits of the House were let loose upon it, and that the somewhat stale jokes of Peter Pindar reared their feeble heads in the mother of Parliaments. The proceeding was not particularly creditable, either to the House itself or to those who exercise control over the order of its proceedings.

The truth is that a well-considered measure of the kind proposed, especially if it dealt with dwellings as well as with persons, would add greatly to the comfort of a considerable section of the poorest classes of the metropolis and other great centres of population. The tenements inhabited by these classes swarm with vermin to an extent which those who have not inspected them could scarcely credit, and the tenants suffer from loss of rest in a corresponding degree. Scores or hundreds of men may be seen, on every fine and warm day, sleeping on the grass in the London parks; and these, or at least a large proportion of them, are persons who cannot sleep at night in their own lodgings for bugs. The chinks of the woodwork in their rooms are often literally choked with bugs, and any clean furniture brought into such places would be invaded before it had rested in them many hours. The skin diseases treated in the metropolitan hospitals are greatly aggravated by lice in the clothes and on the persons of the sufferers; and in the skin department of University College Hospital, as well, probably, as in others, an important part of the treatment consists in sending the clothes of the out-patients to be baked during the time when the wearers themselves are having baths, which, in whatever other way they may be medicated, are at least insecticidal. Such treatment, if we may use the term, seems to be somewhat beneath the dignity of a hospital, and certainly ought not to require the intervention of an out-patient department before it can be applied. We are not fond of the invocation of Hercules, nor of calling in the aid of the State to do for any class of people things which they ought to do for themselves; but we think there can be no doubt that the destruction of vermin is commonly beyond the powers of those who suffer from them. The man or woman who has no change of clothing is absolutely helpless against the pests by which he or she is practically certain to be infested; and, even where a change can be procured, the means of destroying vermin are usually quite beyond the reach of the wearer, unless provided by charity. A proper oven is required for clothing, with a power of estimating the degree of heat which is applied; while the domestic vermin, as distinguished from the personal, require even more expensive treatment. The President of the Local Government Board might do many things less useful than the introduction of a Bill framed to meet the actual needs of the population in these respects, and containing provisions by which Medical Officers of Health and their subordinates were made authorities for the purpose of giving effect to its provisions.

A house infested by bugs is as really a "nuisance," in the accepted sanitary sense of the word, as a house which is undrained or insufficiently supplied with water; and there would be no injustice in compelling the owner to repay to the local Board the cost of remedying such a condition. With regard to personal vermin, the Inspector of Nuisances might be empowered to give an order for the baking of clothes, and for the application of proper remedies to the body of the involuntary entomologist. The prevalence of vermin among a community is an unmitigated evil; and we hope the House of Commons will find time, before very long, to discuss one of the most serious torments of the very poor in a spirit rather of sympathy than of ridicule.

